# Rapid bacterial antibiotic susceptibility test based on simple surface-enhanced Raman spectroscopic biomarkers

**DOI:** 10.1038/srep23375

**Published:** 2016-03-21

**Authors:** Chia-Ying Liu, Yin-Yi Han, Po-Han Shih, Wei-Nan Lian, Huai-Hsien Wang, Chi-Hung Lin, Po-Ren Hsueh, Juen-Kai Wang, Yuh-Lin Wang

**Affiliations:** 1 Department of Internal Medicine, Far Eastern Memorial Hospital, New Taipei City, Taiwan; 2 Department of Traumatology, National Taiwan University Hospital, Taipei, Taiwan; 3 Graduate Institute of Electrical engineering, National Taiwan University, Taiwan; 4 Institute of Microbiology and Immunology, School of Life Science, National Yang-Ming University, Taipei, Taiwan; 5 Institute of Atomic and Molecular Sciences, Academia Sinica, Taipei, Taiwan; 6 Departments of Laboratory Medicine and Internal Medicine, National Taiwan University Hospital, National Taiwan University College of Medicine, Taipei, Taiwan; 7 Center for Condensed Matter Sciences, National Taiwan University, Taipei, Taiwan; 8 Department of Physics, National Taiwan University, Taipei, Taiwan

## Abstract

Rapid bacterial antibiotic susceptibility test (AST) and minimum inhibitory concentration (MIC) measurement are important to help reduce the widespread misuse of antibiotics and alleviate the growing drug-resistance problem. We discovered that, when a susceptible strain of *Staphylococcus aureus* or *Escherichia coli* is exposed to an antibiotic, the intensity of specific biomarkers in its surface-enhanced Raman scattering (SERS) spectra drops evidently in two hours. The discovery has been exploited for rapid AST and MIC determination of methicillin-susceptible *S. aureus* and wild-type *E. coli* as well as clinical isolates. The results obtained by this SERS-AST method were consistent with that by the standard incubation-based method, indicating its high potential to supplement or replace existing time-consuming methods and help mitigate the challenge of drug resistance in clinical microbiology.

Since the discovery of penicillin’s antimicrobial ability by Fleming in 1928, antibiotics have greatly extended human’s lifespan. Antibiotic is hence one of the most important medical inventions in the 20th century and we have taken it for granted in treating many deadly infectious diseases. Right in early epoch had already envisaged antibiotic resistance even before experimental confirmation by Fleming. Nowadays, the overuse and misuse of antibiotics in human and livestock have led to an explosion of antibiotic-resistant bacteria. The growing antibiotic resistance has imperiled our ability to treat patients and is among the top public health menaces in the 21th century. Moreover, during the past decades, multidrug-resistant organisms (MDROs) have become a global emergency. Patients infected with MDROs are associated with higher medical costs, morbidity and mortality. Antibiotic-resistant infections currently claim ~700,000 lives per year in the world. “The continued rise in resistance by 2050 would lead to 10 million people dying per year and a reduction of 2% to 3.5% in gross domestic product,” according to the first annual report by Review on Antimicrobial Resistance^1^. One of the important measures to tackle this problem is fast detection bacterial antibiotic susceptibility for opportunely selecting proper antibiotic treatment.

Existing antibiotic susceptibility test (AST) is based on the following three methods: disk diffusion, gradient diffusion, and agar/broth dilution^2^. Clinical & Laboratory Standards Institute (CLSI) regularly revises the standard protocols of these standard AST methods^3^. The most time-consuming factor common to these methods is the overnight incubation. A number of exciting new techniques have been devised to meet the need for rapid AST^4^. Several noticeable examples are given below. Flow cytometry follows the viability of bacteria by using impermeant dyes after exposure to antibiotics^5^,^6^. Matrix-assisted laser desorption ionization–time of flight mass spectrometry (MALDI-TOF MS)^7^ has been applied to assess antibiotic resistance^8^ by detecting resistance-associated proteins (such as *β*-lactamases), identifying resistant strains, and observing the change in protein mass spectrum in the presence of an antibiotic. Weighing bacteria with and without antibiotic treatment by vibrating cantilevers^9^,^10^ confers the distinction from viable bacteria remained^11^. Isothermal microcalorimetry allows the measurement of heat production from the metabolism of keenly growing bacteria^12^,^13^,^14^. Rotating magnetic ligand-modified beads driven by a revolving magnetic field would change the frequency of rotation by the binding of bacteria. If the bacteria multiplication is influenced by an antibiotic, the rotation frequency change accordingly, indicating the effectiveness of the antibiotic^15^. Micro- and nano-droplets, being small reaction containers^16^, contain bacteria whose viability under antibiotic challenge can be monitored by epifluorescence^17^. A procedure based on real-time PCR has been developed to determine the susceptibility by monitoring pathogenic load with the highly conserved 16S rRNA gene in blood samples exposed to different antimicrobial drugs^18^. Raman scattering has been applied to monitor the changes in the spectral features of enterococci in the absence and presence of vancomycin. Resistant and susceptible strains were differentiated based on an empirical ‘vancomycin effect score’ derived from the changes of bacteria Raman spectra^19^. All of these new techniques are still under development and have not replaced the traditional AST methods.

The recent development of surface-enhanced Raman scattering (SERS) has emerged as a new paradigmatic approach to assay molecules of extremely low concentration^20^. Our team has demonstrated highly sensitive, uniform and reliable SERS substrate based on two-dimensional hexagonally packed Ag nanoparticles embedded in nanochannels of anodic aluminum oxide^21^. We have employed this technique to detect bacteria with high sensitivity^22^, biocides in water^23^, and copper chlorophyll a in vegetable oils^24^. Last, but not least, the detection of bacteria can be carried out while the bacteria are alive. In other words, this technique monitors bacterial activity and simultaneously confers spectroscopic specificity.

In this work, we report the discovery of simple SERS spectroscopic biomarkers for bacterial AST and determination of minimum inhibitory concentrations (MICs). The schematic comparison between the SERS-AST method and the standard broth dilution (BD) method is shown in Fig. 1. They both start with overnight bacterial culture, followed by concentration adjustment of bacteria. The following step of the SERS method is to inoculate solutions of different antibiotic concentrations for 2 hr, while that of the BD method is to inoculate them overnight. The SERS biomarker signal of each solution is then measured for the SERS method. The turbidity level of the solution is alternatively determined for the BD method. In the end, MICs by the methods are read based on the acquired biomarker signal and turbidity level, respectively. The demonstration of the SERS method in AST and determination of MIC were carried out in prototypical *Staphylococcus aureus* (*S. aureus*) and *Escherichia coli* (*E. coli*) as well as clinical isolates of several bacterial species. In this proof-of-concept study, the SERS spectra of methicillin-susceptible *S. aureus* (MSSA) and wild-type *E. coli* treated by oxacillin and imipenem respectively are recorded as a function of treatment time and the decreases in the respective biomarker signals are employed to indicate the effectiveness of the antibiotics. Oxacillin and imipenem both belong to the family of beta-lactam antibiotics, which exerts their antimicrobial activities through binding to the penicillin-binding proteins (PBPs), by which inhibited the cell wall synthesis and cause bacterial death. Oxacillin was chosen because it is the drug of choice for treatment of MSSA infection, while imipenem has been commonly used for patients with severe infections due to Gram-negative bacteria, including *Escherichia coli, Klebsiella pneumoniae, Acinetobacter baumannii,* and *Pseudomonas aeruginosa.* This method has been applied to clinical isolates from a major tertiary hospital using different antibiotics. The results were found to be consistent with the ones obtained by the traditional methods. We also discuss the possible origin of the change in the bacterial SERS spectra in response to the antibiotics treatment and how to comprehend the established criteria for AST and determination of MIC.

## Results

### SERS-based AST

**Figure 2:**
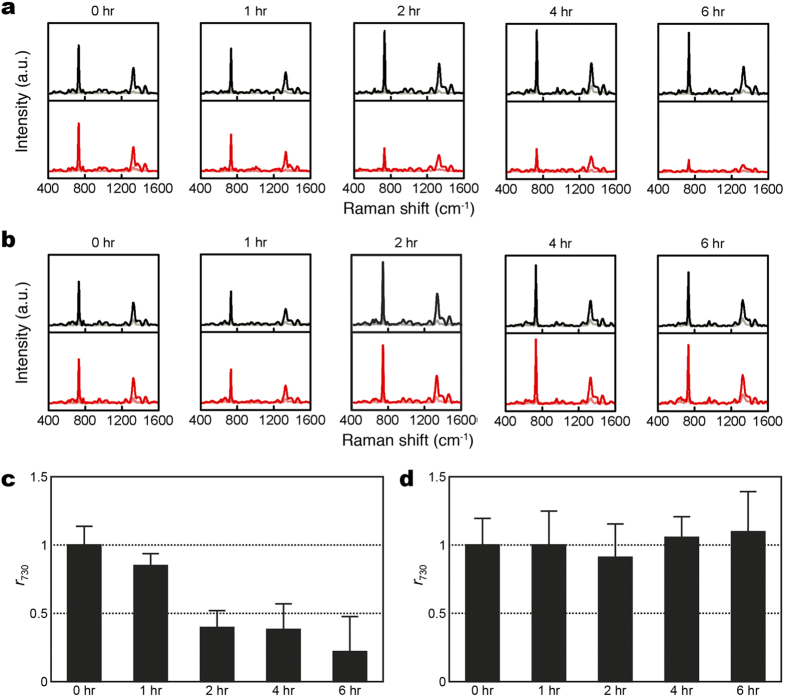
Evolution of SERS spectra for methicillin-susceptible and -resistant *S. aureus* (MSSA and MRSA) with and without oxacillin treatement. (**a**) *S. aureus* (MSSA, ATCC 29213); (**b**) clinical isolate of MRSA with (red curves) and without (black curves) oxacilin treatment; (**c**) signal ratio of the 730-cm^−1^ SERS peak (*r*_730_) of *S. aureus* (ATCC 29213) as a function of oxacillin treatment time; (**d**) *r*_730_ of MRSA as a function of oxacillin treatment time. Black and red curves represent the mean SERS spectra, while gray and light red curves represent their corresponding standard deviation.

**Figure 3:**
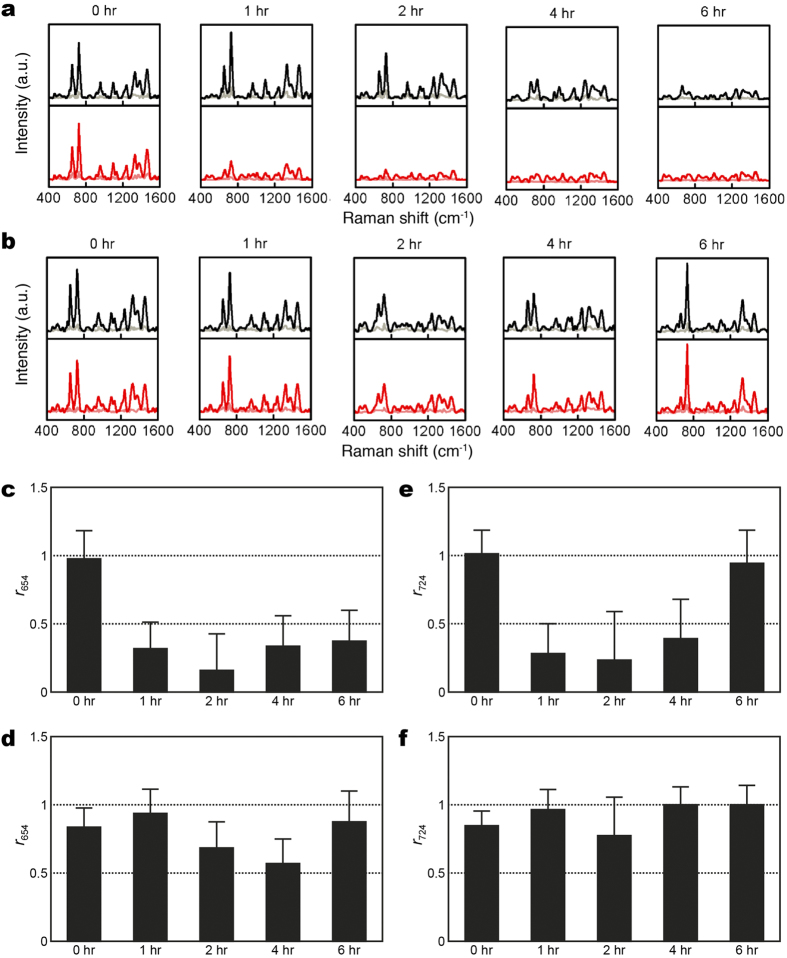
Evolution of SERS spectra for imipenem-susceptible and -resistant *E. coli* with and without imipenem treatment. (**a**) *E. coli* (ATCC 25922); (**b**) clinical isolate of imipenem-resistant *E. coli* with (red curves) and without (black curves) imipenem treatment; (**c**) signal ratio of 654-cm^−1^ SERS peak (*r*_654_) of *E. coli* (ATCC 25922) as a function of imipenem treatment time; (**d**) *r*_654_ of imipenem-resistant *E. coli* as a function of imipenem treatment time; (**e**) signal ratio of 724-cm^−1^ SERS peak (*r*_724_) of *E. coli* (ATCC 25922) as a function of imipenem treatment time; (**f**) *r*_724_ of imipenem-resistant *E. coli* as a function of imipenem treatment time. Black and red curves represent the mean SERS spectra, while gray and light red curves represent their corresponding standard deviation.

The first key question of using SERS for AST of bacteria is whether there is significant difference between the SERS spectrum of a susceptible bacteria strain and that of the resistant one after the same antibiotic treatment. Figure 2 shows the evolution of the SERS spectra of oxacillin-susceptible and clinical methicillin-resistant *S. aureus* of inoculum concentration of 1 × 10^8^ CFU/ml treated with oxacillin. Noticeably, the SERS spectrum of the susceptible *S. aureus* strain treated with oxacillin, while maintaining its spectral pattern over the treatment course, started showing significantly smaller than that without the antibiotic treatment after two hours (Fig. 2a,b). Specifically, the extracted *r*_730_—the signal ratio at 730 cm^−1^ between the antibiotic-treated bacteria and the untreated ones (see Methods for its definition)-of the susceptible *S. aureus* strain decreased to less than 0.5 after 2-hr oxacillin treatment (Fig. 2c); in contrast, *r*_730_ for the resistant strain remained to be approximate unity over the 6-hour oxacillin treatment duration (Fig. 2d). Similarly, Fig. 3 shows the evolution of the SERS spectra of imipenem-susceptible and clinical imipenem-resistant *E. coli* of inoculum concentration of 1 × 10^8^ CFU/ml treated with imipenem. The corresponding difference for the susceptible *E. coli* strain arose when the antibiotic treatment time is 1 hour (Fig. 3a,b). Both the extracted *r*_654_ and *r*_724_—the signal ratios at 654 and 724 cm^−1^, respectively, between the antibiotic-treated bacteria and the untreated ones (see Methods for their definitions)—of the susceptible *E. coli* strain decreased to less than 0.5 after 1-hr imipenem treatment (Fig. 3c,e), while that of the resistant strain remained (Fig. 3d,f). As a final note to Fig. 3d, *r*_724_ of the susceptible *E. coli* strain appears to rise again at the imipenem-treatment times of 4 and 6 hours. It is possibly due to the fact that the SERS signals from the samples without the imipenem treatment gradually diminish in time close to the noise level for this particular case. Three inferences can be deduced from the results above. Firstly, the SERS signal of the susceptible bacteria responds more dramatically to the antibiotic treatment than that of their resistant counterparts. Secondly, the prominent Raman peaks of the bacterial SERS spectra (the 730-cm^−1^ peak for *S. aureus* and the 654- and 724-cm^−1^ peaks for *E. coli*) can be exploited as biomarker signals to identify their antibiotic susceptibility. Thirdly, these two results demonstrate that SERS-AST holds the potential to determine the susceptibility of bacteria to a specific antibiotic in two hours and thus allows for expeditiously providing such vital clinical information.

**Figure 4:**
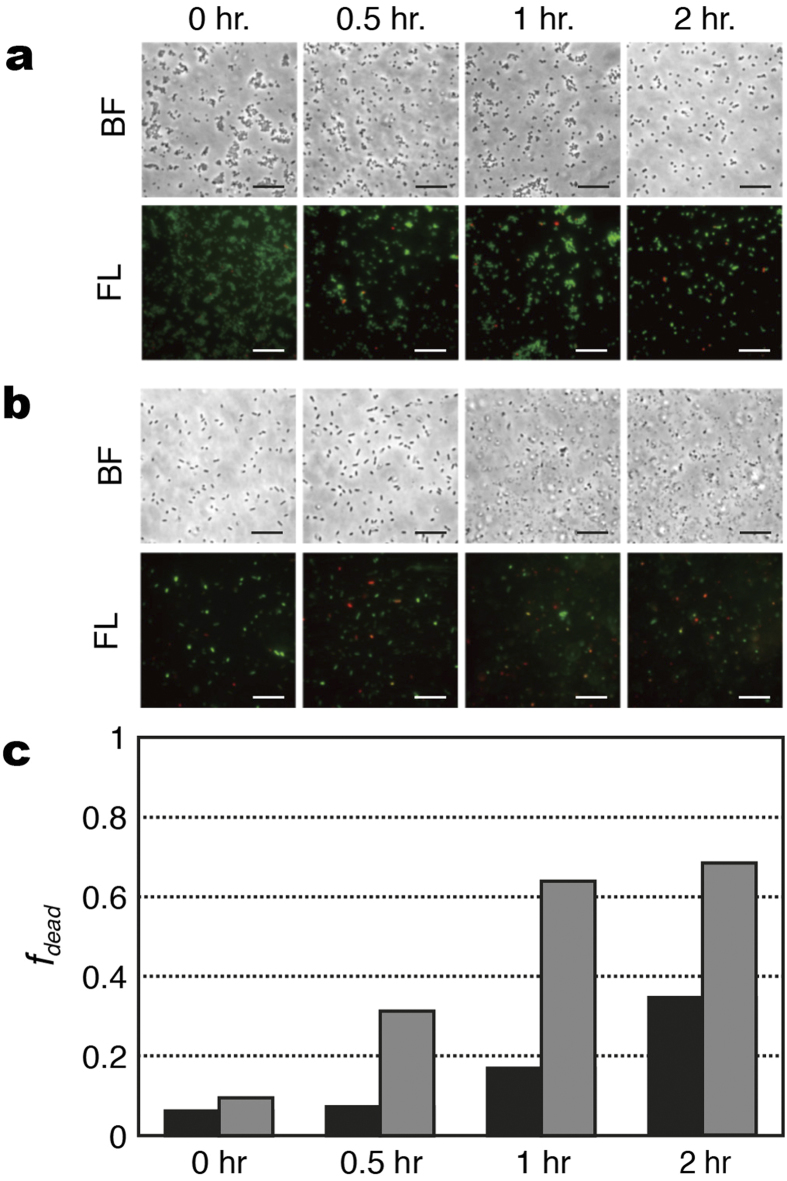
Viability staining tests of Gram-positive and Gram-negative bacteria after antibiotic treatment. After being treated by antibiotics for 0, 0.5, 1 and 2 hours, the bacteria were stained with PI (fluorescing red) and SYTO 9 (fluorescing green). BF: bright-field image; FL: fluorescence image. (**a**) *S. aureus* (ATCC 29213) treated with oxacillin; (**b**) *E. coli* (ATCC 25922) treated with imipenem; (**c**) percentage of dead bacteria (*f*_*dead*_): *S. aureus* (black columns) and *E. coli* (gray columns). *f*_*dead*_ = *N*_*r*_/(*N*_*g*_ + *N*_*r*_ − *N*_*g*+*r*_), where *N*_*r*_ is the count of the red signatures, *N*_*g*_ is the count of the green signatures, and *N*_*g*+*r*_ is the count that the green and red signatures overlap. The scale bars represent 20 *μ*m.

It is interesting to understand the origin of the change in the SERS biomarker signals of the susceptible strains in response to the antibiotic treatment. Such understanding would help put the SERS-AST method on a firmer foundation. One of the key questions to ask is whether the biomarker signal change is related to the viability of bacteria. This could be answered by the viability staining testing of the MSSA and *E. coli* strains after the treatment of oxacillin and imipenem, respectively. The images of the MSSA strain (ATCC 29213) at 0, 0.5, 1 and 2 hr after the oxacillin treatment are shown in Fig. 4a: bright field (BF) and merged fluorescence (FL) images of the bacteria stained with SYTO 9 and PI. Similarly, the corresponding images of the *E. coli* strain (ATCC 25922) at 0, 0.5, 1 and 2 hours after the imipenem treatment are shown in Fig. 4b. The percentage of the damaged cells, *f*_*dead*_ (see Methods for definition), determined from the merged fluorescence images of the two bacteria species at the different times after antibiotic treatment are shown in Fig. 4c. Notice that *f*_*dead*_ of the MSSA strain increases monotonically from 6% to 35% with the antibiotic-treatment time while that of the susceptible *E. coli* strain rises from 10% to 64% at after only one hour of antibiotic treatment, indicating that *S. aureus* respond to oxacillin slower than *E. coli* to imipenem. The SERS biomarker signals of susceptible *S. aureus* and *E. coli* exhibited similar response to the treatment of their respective antibiotics (Figs 2c and 3c,e, respectively). Specifically, it took two hours of oxacillin treatment for *r*_730_ of *S. aureus* to drop below 0.5 while one hour of imipenem treatment was enough to reduce the *r*_654_ and *r*_724_ of *E. coli* below 0.5. Such a strong correlation thus underpins the use of the SERS biomarker signals in the SERS-AST method.

Another question regarding the SERS-AST method is whether the response of the SERS biomarker signals of *S. aureus* and *E. coli* to oxacillin and imipenem, respectively, is equally applicable to the other antibiotics. The results of the MSSA strain (ATCC 29213) after 2 hours of cefoxitin, vancomycin and colistin treatment show that *r*_730_ < 0.5 for cefoxitin and vancomycin while *r*_730_ > 0.5 for colistin. This outcome is consistent with that obtained with the standard AST method: the MSSA strain (ATCC 29213) is susceptible to cefoxitin and vancomycin, but resistant to colistin. Similarly, the corresponding results of the susceptible *E. coli* strain (ATCC 25922) after two hours of ciprofloxacin, colistin and vancomycin treatment show that *r*_654_ and *r*_724_ are less than 0.5 for ciprofloxacin and colistin while *r*_654_ and *r*_724_ larger than 0.5 for vancomycin. These results again agree with that obtained with the standard AST method: the susceptible *E. coli* strain (ATCC 25922) is susceptible to ciprofloxacin and colistin, but resistant to vancomycin. These positive results of the SERS-AST method applied to other antibiotics corroborate its deployment in testing drug susceptibility of bacteria and also incentivize us to explore the possibility of using it to determine the MICs of specific antibiotic for certain bacterial strain.

### Determination of MICs

The progression of the SERS biomarkers—*r*_730_ for *S. aureus* and *r*_654_ and *r*_724_ for *E. coli*-offers the direct quantitative indication of the amount of viable bacteria in a specific environment. Since determining MICs of antibiotics for the susceptible bacterial strains still currently relies on time-consuming culture-based approaches, the SERS-AST method presented in this study provides a new way to detect live bacteria under the antibiotic challenge, qualitatively similar in nature to the culture-based methods, while at a faster speed. According to the time evolution of the bacterial SERS signal with the antibiotic treatment presented above, the 2-hour antibiotic treatment time is long enough to indicate the antibiotic susceptibility. Therefore, only the SERS biomarker signals at the antibiotic treatment times of 0 and 2 hours were compared in the following protocol developed for determining MIC based on the SERS-AST method.

**Figure 5:**
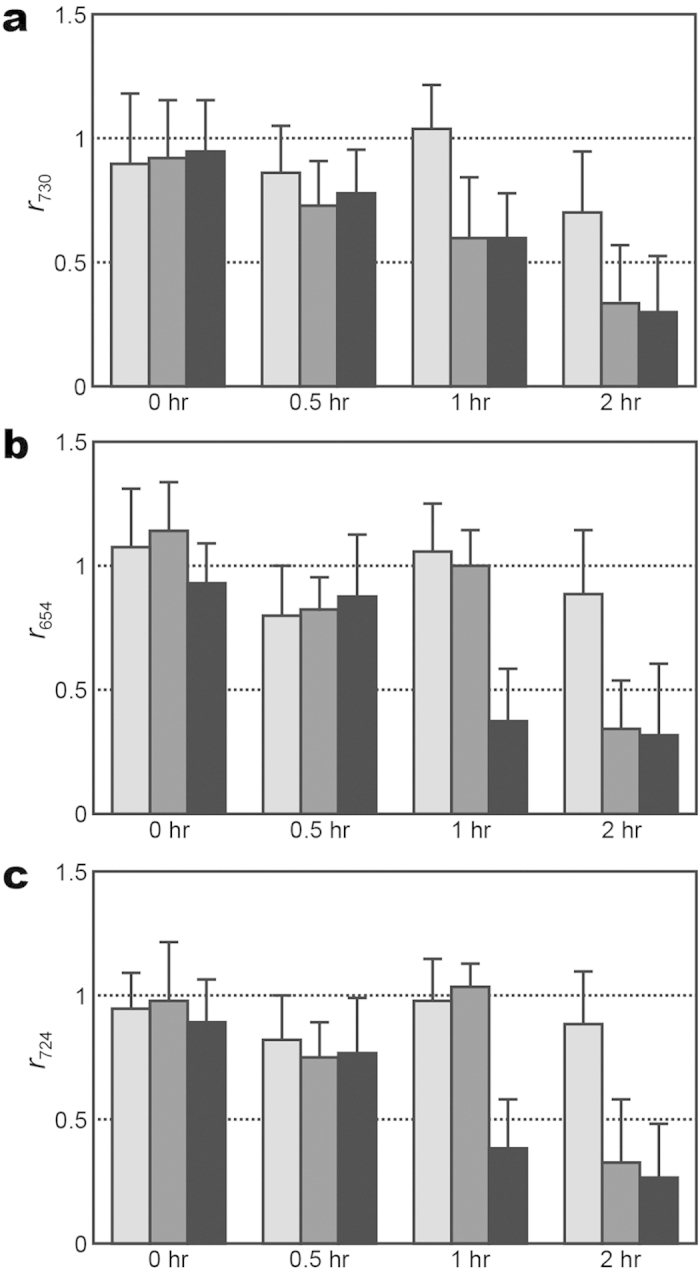
Evolution of SERS biomarker signal versus antibiotic treatment time for different concentrations of antibiotics. (**a**) Signal ratio of 730-cm^−1^ SERS peak (*r*_730_) of *S. aureus* versus vancomycin treatment time at concentrations of 0.5 *μ*g/ml (light gray columns), 1 *μ*g/ml (gray columns), and 2 *μ*g/ml (black columns); (**b**,**c**) signal ratios of 654-cm^−1^ SERS peak (*r*_654_) and 724-cm^−1^ SERS peak (*r*_724_) of *E. coli*, respectively, versus imipenem treatment time at concentrations of 0.03 *μ*g/ml (light gray columns), 0.06 *μ*g/ml (gray columns), and 0.12 *μ*g/ml (black columns).

For 1 × 10^6^ CFU/ml MSSA (ATCC 29213) and *E. coli* (ATCC 35218), their SERS responses to different concentrations of vancomycin and imipenem respectively were scrutinized. The SERS spectrum of the *S. aureus* treated with 1 *μ*g/ml vancomycin exhibited smaller overall intensity than that without the vancomycin treatment (Supplementary Fig. S1a). Similarly, the SERS spectrum of the *E. coli* strain treated with 0.06 *μ*g/ml imipenem exhibited smaller overall intensity than that without the imipenem treatment (shown in Supplementary Fig. S1b). These SERS response can be employed to extract the ratio of the biomarker signals: *r*_730_, *r*_654_ and *r*_724_, which are plotted against the antibiotic treatment time, as shown in Fig. 5. The *r*_730_ of *S. aureus* strain treated with both vancomycin concentrations of 1 and 2 *μ*g/ml drops to below 0.5 at the 2-hr antibiotic treatment, while that with 0.5 *μ*g/ml vancomycin remains above 0.5 after two hours. (Figure 5a). Additionally, *r*_654_ and *r*_724_ of the *E. coli* strain treated with both 0.06 and 0.12 *μ*g/ml imipenem drop to below 0.5 after two hours, while those treated with 0.03 *μ*g/ml imipenem remain above 0.5 (Fig. 5b,c). If the 0.5-break point criterion after two hours of antibiotic treatment is adopted, the MIC value of vancomycin for the *S. aureus* strain was 1 *μ*g/ml and that of imipenem for the *E. coli* strain was 0.12 *μ*g/ml. These MIC values determined by the SERS-AST method were compared with the ones determined by the standard broth microdilution method for the same bacterial concentration of 1 × 10^6^ CFU/ml (Table 1). Note that the two MIC values for the *S. aureus* strain are the same, while that for the *E. coli* strain determined with the SERS-AST method is half of the one determined by the standard broth microdilution method. These results raise an interesting issue concerning the determination of MICs: how do the MIC values determined by the SERS-AST method depend on the inoculum density of bacteria?

**Figure 6:**
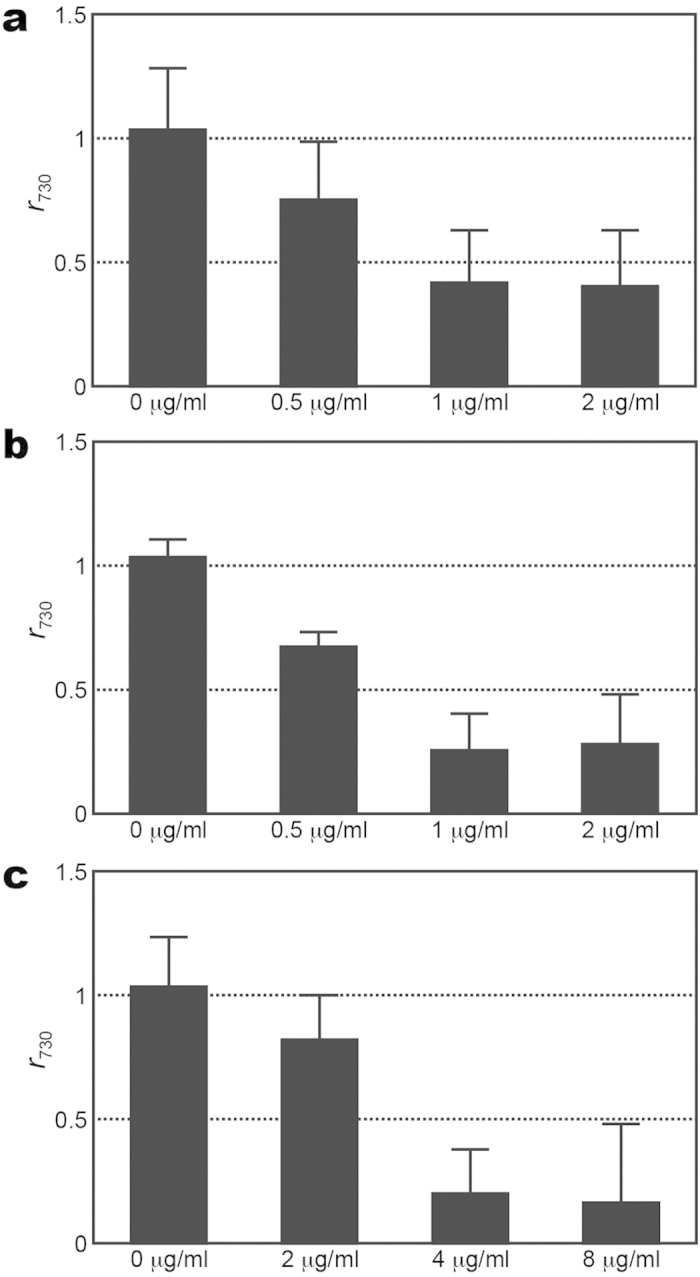
SERS biomarker signals of *S. aureus* versus vancomycin concentration for different inoculum densities. (**a**–**c**) Signal ratio of 730-cm^−1^ SERS peak (*r*_730_) of *S. aureus* of inoculum densities of 10^6^, 10^7^ and 10^8^ CFU/ml, respectively, after being treated with vancomycin of different concentrations for 2 hrs.

**Figure 7:**
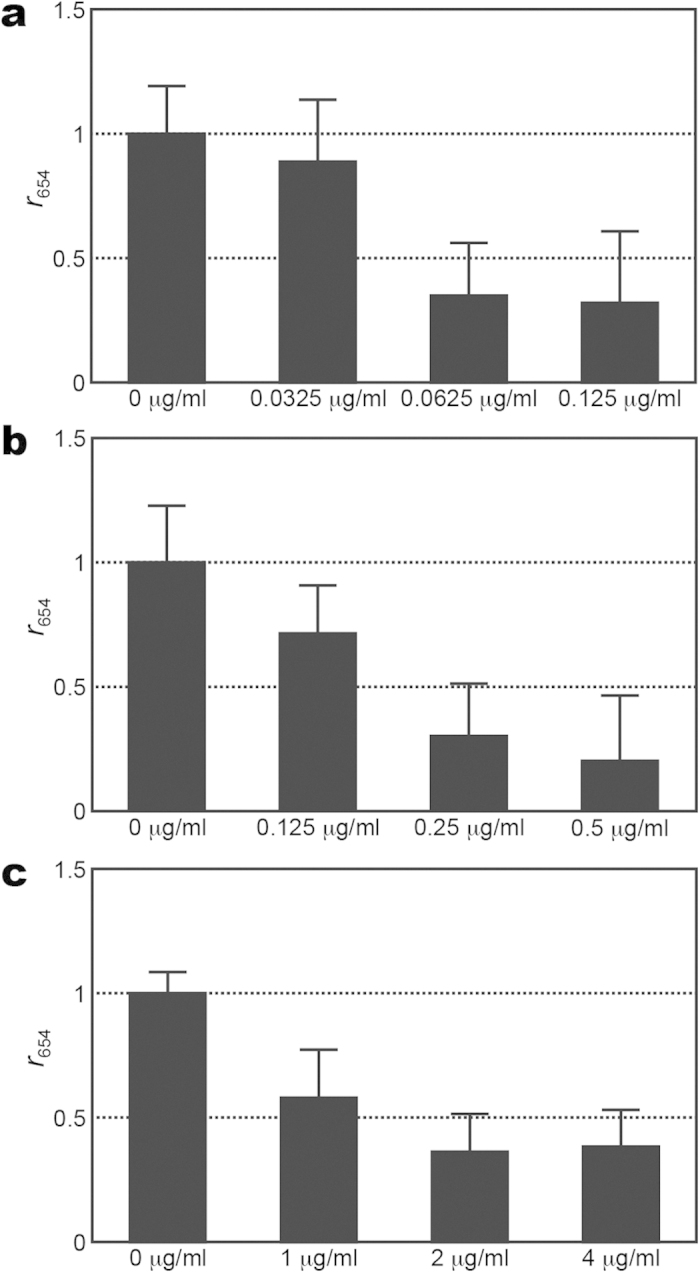
SERS biomarker signals of *E. coli* versus imipenem concentration for different inoculum densities. (**a**–**c**) Signal ratio of 654-cm^−1^ SERS peak (*r*_654_) of *E. coli* of inoculum densities of 10^6^, 10^7^ and 10^8^ CFU/ml, respectively, after being treated with different concentrations of imipenem for 2 hrs.

Two series of SERS spectra of MSSA (ATCC 29213) treated with the different concentrations of vancomycin (0, 0.5, 1 and 2 *μ*g/ml) were acquired using two different bacterial inoculum densities of 1 × 10^6^ and 1 × 10^7^ CFU/ml, while one series of SERS spectra of the same *S. aureus* strain treated with different concentrations of vancomycin (0, 2, 4 and 8 *μ*g/ml) were procured using a bacterial density of 1 × 10^8^ CFU/ml (shown in Supplementary Fig. S2). For each bacterial inoculum density, *r*_730_ was then determined and plotted as a function of the vancomycin concentrations (Fig. 6). Notice that *r*_730_ with the two lower inoculum densities (1 × 10^6^ and 1 × 10^7^ CFU/ml) declines below 0.5 under the treatment of 1 *μ*g/ml vancomycin for two hours, while that with the highest inoculum density (1 × 10^8^ CFU/ml) does not drop below 0.5 until under the treatment of 4 *μ*g/ml vancomycin for two hours. This behavior for the *S. aureus* strain indicates that the inoculum density does influence the outcome when the bacterial concentration reaches 1 × 10^8^ CFU/ml. Similar tests were performed on the wild-type *E. coli* strain (ATCC 35218). Three series of SERS spectra of the *E. coli* strain of three different bacterial density (1 × 10^6^, 1 × 10^7^ and 1 × 10^8^ CFU/ml) were examined after being treated with different concentrations of imipenem (shown in Supplementary Fig. S3): (1) 0, 0.0325, 0.0625 and 0.125 *μ*g/ml for the bacterial density of 1 × 10^6^ CFU/ml; (2) 0, 0.125, 0.25 and 0.5 *μ*g/ml for the bacterial density of 1 × 10^7^ CFU/ml; (3) 0, 1, 2 and 4 *μ*g/ml for the bacterial density of 1 × 10^8^ CFU/ml. As their *r*_654_ and *r*_724_ values are correlated, only the extracted *r*_654_ values were plotted as a function of imipenem concentration for three different bacterial densities in Fig. 7. The MIC values thus determined for the bacterial density of 1 × 10^6^, 1 × 10^7^ and 1 × 10^8^ CFU/ml are 0.0625, 0.25 and 2 *μ*g/ml, respectively. As a consequence, this result for the *E. coli* strain also shows a strong bacterial inoculum density effect.

Table 1 summarizes the MIC results of the MSSA and wild-type *E. coli* strains determined by the SERS-AST method and compares them with the ones determined with the standard broth microdilution method. Two inferences can be drawn from the results. Firstly, the MIC values determined by the broth microdilution method are the same or two times of the ones determined with the SERS-AST method. Secondly, since the SERS-AST method appears more likely to underestimate MIC values for the *S. aureus* and *E. coli* strains in the two higher inoculum densities (1 × 10^7^ and 1 × 10^8^ CFU/ml), a better choice of density for the MIC determination is 1 × 10^6^ CFU/ml for both Gram-positive and Gram-negative bacteria. As a consequence, we chose 1 × 10^6^ CFU/ml as the inoculum density to determine the AST and MIC of clinical bacterial isolates by the SERS-AST method.

### AST and MIC of clinical samples

In the study of clinical isolates, nine isolates of MRSA and 10 each of *E. coli, A. baumannii,* and *K. pneumoniae* were used. Both the SERS-AST method and the standard agar dilution method^3^ were employed to conduct AST and determine MIC values. As shown in Table 2, for nine clinical isolates of *S. aureus*, the MIC values determined by the SERS-AST method are consistent with the ones determined by the standard agar dilution method in six of 9 clinical isolates, while the MIC values of the rest three clinical isolates determined by the SERS-AST method are only one dilution different from the ones determined by the standard agar dilution method. According to the latest CLSI guidelines breakpoints^3^, all the susceptibility results from the two methods are considered in good agreement. For clinical isolates of *E. coli*, four of the 10 *E. coli* isolates have their MIC values one dilution lower than that obtained with the standard agar dilution method, one isolate has a MIC value two dilutions lower, and the MIC value of one isolate is two dilutions higher. Both methods determined the same MIC values in other four isolates of *E. coli.* Despite of the difference of MIC values, seven of 10 strains had consistent susceptibility results, while only 1 isolate was misclassified as susceptible to imipenem when it was actually intermediately resistant. For clinical isolates of *A. baumannii*, six out of 10 isolates have the same MIC data obtained by the two methods, while the MIC values in four isolates determined with the SERS-AST method are one dilution lower. Only two isolates of *A. baumannii* was misclassified as susceptible to imipenem when it was actually intermediately resistant. In spite of these differences shown in the bacterial species above, the susceptibility interpretations are the same according to the most recently updated CLSI guidelines^3^. No major error was found. Finally, in contrast to *S. aureus, E. coli* and *A. baumannii*, the MIC results of clinical isolates of *K. pneumoniae* determined with the SERS-AST method have a higher discrepancy rate. Only five of the 10 isolates of *K. pneumoniae* have the same MIC values reported by both the methods. The MIC values of three isolates determined with the SERS-AST method are one dilution lower than that determined with the standard agar dilution method, while two isolates bear the MIC values that are two dilutions lower. Only one isolate of *K. pneumoniae* was misclassified as intermediately resistant to imipenem when it was actually resistant. Again, there is no major discrepancy noted according to the current CLSI breakpoint guidelines^3^. Table 3 summarizes the comparison in MICs of these four bacterial species performed with the SERS-AST method and the standard agar dilution method. The MIC values determined with the SERS-AST method are generally the same or one dilution different from the ones determined with the traditional agar dilution method. Only one isolate of *E. coli* and two isolates of *K. pneumoniae* had two-dilution difference. Nevertheless, most of these discrepancies did not affect the susceptibility interpretation and no major error was found.

## Discussion

We have empirically demonstrated the validity of the SERS-AST method for rapid bacterial AST and MIC determination. In order to put the method on a firmer foundation, it is helpful to better understand the molecular origins of the SERS vibrational s at 730 cm^−1^ and 654/724 cm^−1^ for *S. aureus* and *E. coli*, respectively, though they are still under investigation. One possible candidate is certain molecular constituent of the bacteria cell wall^22^ such as peptidoglycan, which is supposed to be in close contact with the SERS substrate. Since the SERS-active region extends only a few nanometers above the surface of Ag nanoparticles^25^, presumably some vibrational bands of such cell-wall molecules would be shown in the SERS of bacteria. However, this hypothesis appears to be inconsistent with the observation that bacterial SERS spectra were found to be different from the SERS spectra of major constituent molecules of bacteria cell-wall including *N*-acetylglucosamine and *N*-acetylmuramic acid. Additional evidence against this hypothesis is the observation described above that the intensities of the biomarker signals exhibit strong correlation with the number of viable bacteria in the assay. Although not entirely impossible, it seems unlikely that the cell walls of dead bacteria would contain less amount of the molecule responsible for the biomarker and lead to the decrease in the SERS biomarker signals. Apparently, more researches are entailed to completely rule out this hypothesis that certain cell-wall molecules are responsible for the SERS biomarker signals.

The other possible candidates are certain metabolic molecules released from live bacteria. Based on this proposition, one would expect drug-susceptible bacteria to be either killed or forced to change their metabolic process, resulting in the reduction in SERS biomarker signals. On the other hand, the number of drug-resistant bacteria under the same antibiotic treatment would remain constant and their metabolites would be expected to discharge normally. Qualitatively, these expectations agree with the results obtained with the SERS-AST method (Figs 2 and 3) and the viability-staining test (Fig. 4) described above. Furthermore, this hypothesis is also consistent with the report that the SERS spectra of supernatants from washed and spun bacteria also exhibited a strong ~730 cm^−1^ peak^26^. That is, the molecules responsible for this peak appear to be detached from the bacteria and therefore were found in the supernatants. The detachment of these molecules from the bacteria is further supported by the observation that the strong ~730 cm^−1^ peak appears not only in the SERS spectra acquired from areas covered by *S. aureus* but also from the empty areas (Fig. S4). Reference 26 speculated that the detached molecules responsible for this strong SERS peak ‘is probably due to capsular polysaccharides in the supernatant’. We think it is unlikely because the SERS of saccharides do not exhibit a strong peak near ~730 cm^−1^ 27.

The molecule responsible for the 730 cm^−1^ peak of *S. aureus* is more likely to be adenine and/or its derivatives with a prominent peak near 730 cm^−1^ while that for the 654 and 724 cm^−1^ peak of *E. coli* are likely guanine, hypoxanthine and/or their derivatives, respectively. The reasons for this tentative assignment are two folds. Firstly, Rinas and coworkers showed that purine derivatives are constantly excreted from live bacteria^28^. Secondly, the noticeable 730-cm^−1^ peak in the SERS spectrum of *S. aureus* coincides with that the corresponding one in the SERS spectrum of adenine^29^, while the two palpable peaks at 654 and 724 cm^−1^ in the SERS spectrum of *E. coli* are in good agreement of the corresponding ones in the SERS spectra of guanine^30^ and hypoxanthine^31^, respectively. Therefore, the decrease of the SERS biomarkers at 730 and 654/724 cm^−1^ in response to the antibiotic treatment is presumably a manifestation of the disrupted purine metabolism during the dying process of susceptible bacteria.

If the SERS biomarker signals indeed originate from certain bacterial metabolic molecules, it is interesting to speculate on the metabolic pathway that allows their release to the environment. For examples, massive gene expression in bacteria could be triggered by environmental stress such as antibiotic exposure. In clinical isolates of vancomycin-treated *S. aureus,* it has been demonstrated that genes associated the housekeeping functions or cell-wall synthesis were obviously activated after antibiotic exposure^32^. Similarly, in *A. baumannii* exposed to heat shock or different kinds of antibiotics, including ampicillin plus sulbactam, cefepime, meropenem, and sulfamethoxazole/trimethoprim, a chaperon gene, *DnaK*, is up-regulated as well^33^. It is noteworthy that these antibiotics exert their anti-bacterial activities through different mechanisms, including cell wall synthesis inhibition and folate metabolism inhibition. It is possible that a common metabolic pathway is induced or interrupted when bacteria are exposed to a variety of stress. This speculation appears consistent with our observation that similar change of SERS spectra occurred when the bacteria were treated with antibiotics based on different antimicrobial mechanisms, including beta-lactam agents, vancomycin and fluoroquinolones, which exert their antimicrobial actions through cell-wall inhibition and DNA gyrase inhibition respectively. Certainly, further researches are necessary to verify these speculations. However, we would like to note that, if certain bacterial metabolites were indeed the origin of the bacterial SERS biomarker signal, the SERS-AST method would become a new method to detect trace of metabolites^34^,^35^, and therefore it would have high potential for studying the metabolic activity of bacteria in *ex vivo* conditions (*e.g*., under antibiotic challenge).

The choices of the two-hour antibiotic treatment time for the SERS-AST method was based on the observation that the SERS biomarker signals from susceptible strains of bacteria dropped below 0.5 after two hours of treatment by a specific antibiotic at a concentration above MIC. (Figs 2c,d and 3c–e). The choices were purely empirical, i.e., they were derived from the good agreement reached between the MICs determined with the SERS-AST method and those with the standard agar dilution method (Table 2). Such agreement would not have been possible without the highly uniform and reliable SERS-substrates used in our study. It is conceivable to adopt a shorter treatment time, such as one hour, by choosing a higher break point of the SERS biomarker signal ratio and using an optimized antibiotic treatment conditions.

In order to show the general validity of the SERS-AST method, there are several issues need to be addressed. Firstly, we need to test if other clinically relevant Gram-positive species also have the 730 cm^−1^ peak and Gram-negative species have 654 and 724 cm^−1^. As shown by the data in Fig. S5, the SERS spectra of many Gram-positive bacteria indeed exhibit the prominent 730 cm^−1^ peak and that many Gram-negative species have the 654 and 724 cm^−1^ peaks. Although the number of species studied is significant but still not enough to draw the conclusion. Secondly, it is necessary to test the response of these SERS signals from other clinically relevant species under the attack by antibiotics. Thirdly, it would be helpful if the SERS-AST method can be shown to be applicable to samples with a mixture of more than two bacteria species.

Although it is too early in this demonstration of principle study to compare the SERS-AST method with other methods under development, it is worth pointing out some of its advantages, such as the time required to determine the antibiotic susceptibility and MIC of a bacteria strain in response to certain antibiotic is shorter than that required by the standard culture-based methods^36^, which usually call for overnight incubation. Another advantage is that the SERS-AST method is derived from the response of viable bacteria to antibiotics, similar to the standard culture-based methods. This similarity is expected to provide more direct correlation between the two methods than other methods, such as mass spectrometry that derives the correlation by analyzing the constituents of dead bacteria^8^.

In summary, the intensities of specific biomarkers in the SERS spectra of a susceptible strain of *S. aureus* or *E. coli* were discovered to drop evidently in two hours when the bacteria were exposed to antibiotics. The drop of the biomarker signals has been exploited for rapid AST and MIC determination of the MSSA and wild-type *E. coli* as well as clinical isolates. A protocol based on SERS for bacterial AST and determination of MICs for certain antibiotic was established. The results obtained with the SERS-AST method on clinical isolates were in good agreement with the ones obtained with the traditional methods and no major error was found according to CLSI guidelines. In comparison to many other methods under development, this new rapid method offers a unique advantage of conducting AST on viable bacteria, which allows the quantitative determination of MIC at the same time, similar to the standard culture-based methods. Its ability to provide such critical information about the bacteria affecting a patient in a few hours could help mitigate the rising challenge of drug-resistant bacteria strains.

## Methods

### Bacterial samples

Methicillin-susceptible *Staphylococcus aureus* (ATCC 29213) and *Escherichia coli* (ATCC 25922 and ATCC 35218) were obtained from American Type Culture Collection (ATCC). A total of 39 clinical isolates of *A. baumannii* (*n* = 10)*, K. pneumoniae* (*n* = 10)*, E. coli* (*n* = 10), and methicillin-resistant *S. aureus* (MRSA, *n* = 9), including three vancomycin-intermediately-susceptible isolate (VISA) were used. These isolates were randomly selected and were all recovered from patients with bacteremia who were treated at National Taiwan University Hospital. Each of these bacteria was grown in 2 ml trypticase soy broth (TSB) for 16–18 hours at 37 °C, then sub-cultured for 1 hr by adding 0.2 ml of the overnight-cultured sample to 5 ml TSB. The inoculum was diluted to a designated density ranging from 1 × 10^6^ to 1 × 10^8^ CFU/ml based on its optical density at 600 nm—For example, it reads 0.5 for a 1 × 10^8^ CFU/ml sample. Before SERS examination, the bacterial samples were put through a washing procedure to remove incubation media or antibiotics: (1) 1 ml sample was centrifuged at 10000 × g for 2 min at room temperature; (2) its 0.9 ml supernatant part was then replaced by the same amount of sterile water. The washing procedure was then repeated twice, mounting the bacterium-washing protocol. For the SERS examination, each sample was centrifuged at the same condition again and its supernatant part was removed, followed by being diluted to 1 × 10^9^ CFU/ml.

### SERS-active substrate

The fabrication procedure of the SERS-active substrates used in this study was reported previously^21^. Briefly, a glass slide was coated with a 100 nm Al film by sputtering. The Al-coated glass slide was then anodized in sulfuric acid under a bias voltage to create a two-dimensional hexagonally packed array of nanochannels with an average inter-channel spacing of about 50 nm. The nanochannels were further etched chemically to yield an average channel diameter of 45 nm, resulting in a mean gap of 5 nm between adjacent nanochannels. Ag nanoparticles were then grown into the nanochannels via silver electrochemical plating to produce an array of Ag nanoparticles with an average length of 60 nm. Individual SERS-active substrates were then cleaned by rinsing with deionised water, followed by being vacuum-sealed in plastic bags for storage. To minimize surface contamination, each substrate was freshly used immediately after the bag was unsealed. The detailed structural characterizations with scanning electron microscopy (SEM) and transmission electron microscopy (TEM) as well as SERS performance of this substrate were described previously^21^. In short, both top-view SEM and cross-sectional TEM examinations showed both the variations of particle diameter and interparticle gap to be ~2 nm; the SERS signal of rhodamine 6G on this substrate exhibited a highly repeatable correlation with molecular concentration from 10^−9^ to 10^−6^ M; and, lastly, the SERS signal of adenine increased dramatically as the interparticle gap was smaller than 10 nm, indicating the presence of ‘hot junctions’ within the Ag nanoparticle array of this substrate. Moreover, several fundamental studies were undertaken to investigate the electromagnetic resonance traits of this substrate. Firstly, a far-field scattering spectroscopy study was performed and a dipole-coupling model was developed to investigate the plasmon behavior of this substrate as a function of the interparticle gap between Ag nanoparticles of this substrate^37^. Secondly, a high-precision electrodynamic numerical solver based on pseudo-spectral time-domain method was developed to investigate local enhanced field in the interparticle gap—‘hot junction’—of this substrate^38^. Thirdly, a scattering-type near-field optical microscope with sub-10 nm resolution was used to directly observe the field amplitude and phase within the ‘hot junctions’^39^ of the nanoparticle array.

### SERS measurement and spectral processing

SERS measurements were performed with a Raman microscope (HR800, Horiba). A HeNe later emitting at 632.8 nm served as the excitation source. The laser beam, after passing through a laser-line filter to remove residual plasma lines, was focused by a 20× objective lens to the substrate surface on which 1 *μ*l of the bacterial suspension (1 × 10^9^ CFU/ml) was placed and a circle of 2–3 mm in diameter was then formed after 10–20 min. air-drying. The typical laser irradiation power density at the sample surface was about 1 × 10^5^ mW/cm^2^. The scattered radiation was collected backward by the same objective lens and sent through a long-pass filter to an 80-cm monochromator plus liquid-nitrogen cooled charge-coupled device for spectral recording. The integration time of the Raman signal varied from 1 to 3 sec. For each bacterial circular region on the SERS substrate, Raman measurements were performed at several randomly chosen spots. Special attention was paid to prevent any other laser-induced effects such that the spectral patterns thus obtained were stable during signal accumulation and the signal strength was linearly related to the irradiating laser power and the integration time. After the occasionally occurred spectra that exhibited dissimilar spectral patterns or excessively large background were removed, the remaining spectra underwent baseline removal and averaging. The algorithm used for baseline removal of the measured SERS spectra is called sensitive nonlinear iterative peak clipping algorithm^40^. The baseline-removed spectra were then processed to obtain their mean spectrum and the associated standard variation. To be noted, the detection limit is on the order of 1X10^5^ CFU/ml based on the current protocol and instrument.

### AST

The bacteria, after undertaking the bacterium-washing protocol, were diluted to a targeted concentration. Both isolates were challenged with or without a designated antibiotic for a designated duration. The antibiotics used in this study (oxacillin, imipenem, vancomycin, cefoxitin, colistin and ciprofloxacin) were purchased from Sigma-Aldrich. The acquired SERS spectra of the resultant bacterial solutions were analyzed in accordance with the aforesaid signal-processing procedure. The most prominent peak in the SERS spectrum at 730 cm^−1^ was employed to reflect its presence in the sample and the height of the 730-cm^−1^ peak of the bacterial sample—the SERS biomarker signal of Gram-positive bacteria—treated with the antibiotic were compared with that of the bacteria without the antibiotic challenge, yielding the signal ratio at 730 cm^−1^—namely, 
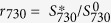
, where 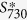 (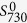) is the signal of the Raman peak at 730 cm^−1^ of the bacterial sample with (without) the antibiotic challenge. The similar protocol was applied to the case of Gram-negative bacteria with another selected antibiotic, except that both their two conspicuous Raman peaks at 654 and 724 cm^−1^—the SERS biomarker signals of Gram-negative bacteria—were analyzed, yielding both *r*_654_ and *r*_724_. Bacteria were prepared with the aforementioned protocol. A designated antibiotic was used to challenge the bacteria for durations of 0, 1, 2, 4, and 6 hours. The corresponding control groups were not pre-treated with any antibiotic. The breaking point of antibiotic-susceptible for Gram-positive bacteria was *r*_730_ < 0.5 with 2-hr antibiotic treatment, while the corresponding one for Gram-negative bacteria was when either *r*_654_ < 0.5 or *r*_724_ < 0.5 was satisfied.

### Determination of MICs

**Figure 8:**
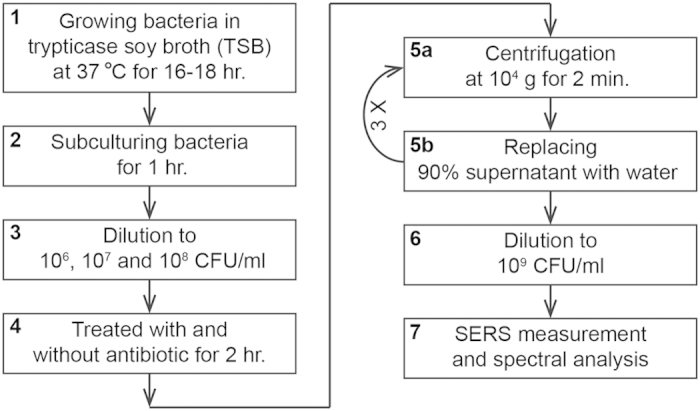
Flow chart of AST and MIC determination. The whole procedure is composed of seven steps (**1** to **7**). Among all these steps, only Step **5** (separated into Step **5a** and **5b**) is repeated three times (indicated with 3×).

Following the protocol for antibiotic susceptibility testing, bacteria were treated with different antibiotic concentrations. The criterion for the determination of MIC was set at the antibiotic concentration at which *r*_730_ < 0.5 for Gram-positive bacteria and *r*_654_ < 0.5 or *r*_724_ < 0.5 for Gram-negative bacteria. The antibiotic-treatment duration that conferred the MIC values most agreed with the standard values were adopted in the study. With these optimized antibiotic-treatment durations determined for the bacterial concentration of 1 × 10^8^ CFU/ml, the SERS protocol to determine the MIC values was repeated for the other two bacterial concentrations of 1 × 10^6^ and 1 × 10^7^ CFU/ml to test their dependence on inoculum size, thus yielding the optimized bacterial concentrations for the antibiotic treatment. The concentrations of antibiotics used in the determination of MIC of any of the clinical isolates were composed of the one determined by standard method (C*) together with higher and lower concentrations. For *S. aureus*, the concentrations of vancomycin were 0.5 × C*, C* and 2 × C*. For *E. coli* and *K. pneumonia*, the concentrations of imipenem were 0.125 × C*, 0.25 × C*, 0.5 × C*, C* and 2 × C*. Finally, for *A. baumannii*, the concentrations of imipenem were 0.25 × C*, 0.5 × C*, C* and 2 × C*. Figure 8 shows the flow chart of AST and determination of MIC.

### Viability staining

The MSSA strain (ATCC 29213) and wild-type *E. coli* strain (ATCC 25922) were grown in 2 ml TSB for 16–18 hours at 37 °C, then sub-cultured for 1 hr. The inoculum was diluted to 1 × 10^8^ CFU/ml. *S. aureus* and *E. coli* were challenged with oxacillin (8 *μ*g/ml) and imipenem (8 *μ*g/ml) for 0, 0.5, 1 and 2 hr, respectively. The samples then underwent the bacterium-washing protocol described above. Finally, the diluted specimens were stained with SYTO 9 (5 *μ*M) and propidium iodide (PI) (30 *μ*M) for 20 min., and then washed 3 times with sterile water. Both dyes were purchased from Life Technologies. The washed specimen were fixed with same volume of 3.7% formaldehyde for 15 min., and then washed with sterile water twice. 10 *μ*l of bacterial suspension was placed on a glass slide and air-dried. The dried specimens were added with 10 *μ*l of mounting medium, and examined with a fluorescence microscope. SYTO 9 fluoresces at ~500 nm and is a cell-permeant nucleic acid stain that can be used to stain RNA and DNA in both live and dead cells, while PI fluoresces at ~620 nm and is a membrane impermeant dye that is generally excluded from viable cells. As results, the green feature in the acquired fluorescence images signifies both live and dead bacteria, while the red feature denotes only dead bacteria. The percentages of the dead bacteria from the fluorescence images—*f*_*dead*_ = *N*_*r*_/(*N*_*g*_ + *N*_*r*_ − *N*_*g*+*r*_), where *N*_*r*_ is the count of the red signatures, *N*_*g*_ is the count of the green signatures, and *N*_*g*+*r*_ is the count that the green and red signatures overlap—were determined at the durations of 0, 0.5, 1 and 2 hours.

### Standard AST of clinical isolates

One species of Gram-positive clinically isolated bacteria (9 isolates of *S. aureus*) and three species of Gram-negative clinically isolated bacteria (10 isolates of *E. coli*, 10 isolates of *A. baumannii*, and 10 isolates of *K. pneumoniae*) of total 39 clinical isolates were collected for the AST of vancomycin and imipenem, respectively. Both the tests with the SERS-AST method and the standard agar dilution method were performed. For the test with the standard method, solutions containing antibiotic were added on the top of agarose, allowing for drug diffusion towards the bacteria. The breakpoint of each antimicrobial agent for the standard method was in accordance with the latest guideline of CLSI^3^. The MIC values determined with the two methods were compared for each antibiotic agent.

## Additional Information

**How to cite this article**: Liu, C.-Y. *et al*. Rapid bacterial antibiotic susceptibility test based on simple surface-enhanced Raman spectroscopic biomarkers. *Sci. Rep.* **6**, 23375; doi: 10.1038/srep23375 (2016).

## Supplementary Material

Supplementary Information

## Figures and Tables

**Figure 1:**
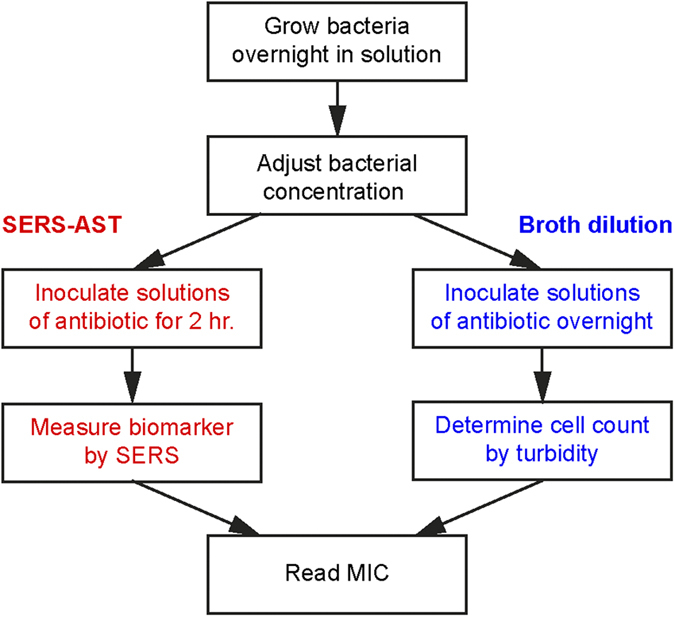
Schematic comparison between the SERS-AST method and the standard broth dilution method.

**Table 1:** Minimal inhibitory concentration (MIC) of vancomycin for *S. aureus* (ATCC 29213) and imipenem for *E. coli* (ATCC 35218) determined by SERS-AST (SERS) method and standard broth microdilution (BMD) method with different inoculum densities.

Bacterial strain	Inoculum density (CFU/ml)	MIC (*μ*g/ml)	
		SERS	BMD
*S. aureus* (ATCC 29213)	1 × 10^6^	1	1
	1 × 10^7^	1	2
	1 × 10^8^	4	8
*E. coli* (ATCC 35218)	1 × 10^6^	0.06	0.12
	1 × 10^7^	0.25	0.5
	1 × 10^8^	2	2

**Table 2:** Minimal inhibitory concentration (MIC) and antibiotic susceptibility testing (AST) of vancomycin in nine clinical isolates of *S. aureus* as well as imipenem in ten clinical isolates of *E. coli*, ten clinical isolates of *A. baumannii*, and ten clinical isolates of *K. pneumoniae* determined by SERS-AST (SERS) method and the standard agar dilution (AD) method with inoculum density of 1 × 10^6^ CFU/ml.

Isolate	MIC (*μ*g/ml) and AST							
	*S. aureus*		*E. coli*		*A. baumannii*		*K. pneumoniae*	
	SERS	AD	SERS	AD	SERS	AD	SERS	AD
1	0.5 (S)	0.5 (S)	2 (I)	4 (R)	2 (S)	2 (S)	2 (I)	2 (I)
2	1 (S)	0.5 (S)	2 (I)	2 (I)	2 (S)	2 (S)	0.5 (S)	2 (I)
3	0.5 (S)	1 (S)	1 (S)	2 (I)	2 (S)	4 (S)	1 (S)	1 (S)
4	1 (S)	1 (S)	2 (I)	4 (R)	2 (S)	4 (S)	1 (S)	1 (S)
5	2 (S)	2 (S)	16 (R)	32 (R)	4 (S)	8 (I)	0.25 (S)	0.5 (S)
6	2 (S)	2 (S)	1 (S)	1 (S)	4 (S)	8 (I)	0.125 (S)	0.5 (S)
7	4 (I)	4 (I)	1 (S)	0.5 (S)	16 (R)	16 (R)	8 (R)	8 (R)
8	4 (I)	4 (I)	0.25 (S)	1 (S)	16 (R)	16 (R)	4 (R)	4 (R)
9	8 (I)	4 (I)	0.5 (S)	0.5 (S)	32 (R)	32 (R)	2 (I)	4 (R)
10	—	—	32 (R)	32 (R)	32 (R)	32 (R)	4 (R)	8 (R)

S: susceptible; I: intermediate; R: resistant.

**Table 3:** Minimum inhibitory concentrations (MICs) of vancomycin for clinical isolates of *S. aureus* and imipenem for clinical isolates of *E. coli, A. baumannii* and *K. pneumonia* determined by SERS-AST method compared with those determined by standard agar dilution method.

MIC Results	Bacterial strain			
	*S. aureus*	*E. coli*	*A. baumannii*	*K. pneumoniae*
Concordant	6	4	6	5
One-dilution discordant	3	5	4	3
Two-dilution discordant	0	1	0	2
