# Supplementary material for: Rapid bacterial antibiotic susceptibility test based on simple surface-enhanced Raman spectroscopic biomarkers
Source: Sci Rep. 2016 Mar 21;6:23375. doi: 10.1038/srep23375 (PMC4800312; doi:10.1038/srep23375)

**Rapid bacterial antibiotic susceptibility test based on simple
surface-enhanced Raman spectroscopic biomarkers**

Chia-Ying Liu^1*^, Yin-Yi Han^2*^, Po-Han Shih^3*^, Wei-Nan Lian^3^, Huai-Hsien Wang^4^, Chi-Hung Lin^3^, Po-Ren Hsueh^5^, Juen-Kai Wang^4,6^, Yuh-Lin Wang^4,7^

^1^Department of Internal Medicine, Far Eastern Memorial Hospital, New Taipei City, Taiwan, ^2^ Department of Traumatology, National Taiwan University Hospital, Taipei, Taiwan ^3^Institute of Microbiology and Immunology, School of Life Science, National Yang-Ming University, Taipei, Taiwan, ^4^Institute of Atomic and Molecular Sciences, Academia Sinica, Taipei, Taiwan, ^5^Departments of Laboratory Medicine and Internal Medicine, National Taiwan University Hospital, National Taiwan University College of Medicine, Taipei, Taiwan, ^6^Center for Condensed Matter Sciences, National Taiwan University, Taipei, Taiwan, ^7^Department of Physics, National Taiwan University, Taipei, Taiwan

Correspondence and requests for materials should be addressed to P. R. H. ([hsporen@ntu.edu.tw); J](mailto:hsporen@ntu.edu.tw); J). K. W. (jkwang@ntu.edu.tw); and Y. L. W. (ylwang@pub.iams.sinica.edu.tw)

* These authors contributed equally to this work.

**Supplementary Figure S1 | Evolution of SERS spectra versus antibiotic concentration.** (**a**) SERS spectra of *S. aureus* (ATCC 29213) treated with different concentrations of vancomycin at 0 and 2 hr.; (**b**) SERS spectra of *E. coli* (ATCC 35218) treated with different concentrations of imipenem at 0 and 2 hr. Black and red curves represent the mean SERS spectra, while gray and light red curves represent their corresponding standard deviation.


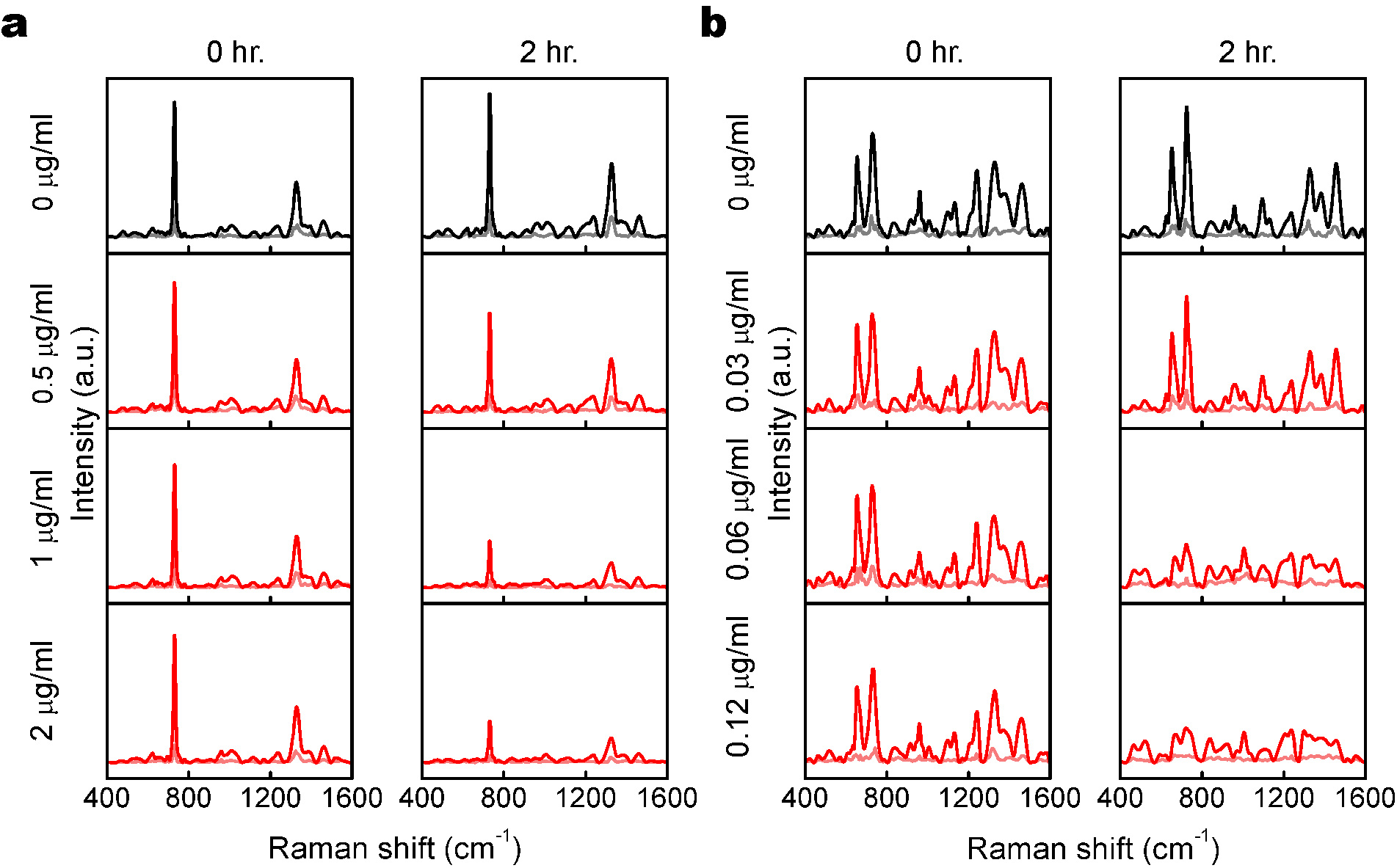


**Supplementary Figure S2 | Evolution of SERS spectra of *S. aureus* versus vancomycin concentration under different inoculum densities.** SERS spectra of *S. aureus* (ATCC 29213) of inoculum densities of 10^6^, 10^7^ and 10^8^ CFU/ml treated with different vancomycin concentrations for 2 hr. Black and red curves represent the mean SERS spectra, while gray and light red curves represent their corresponding standard deviation.


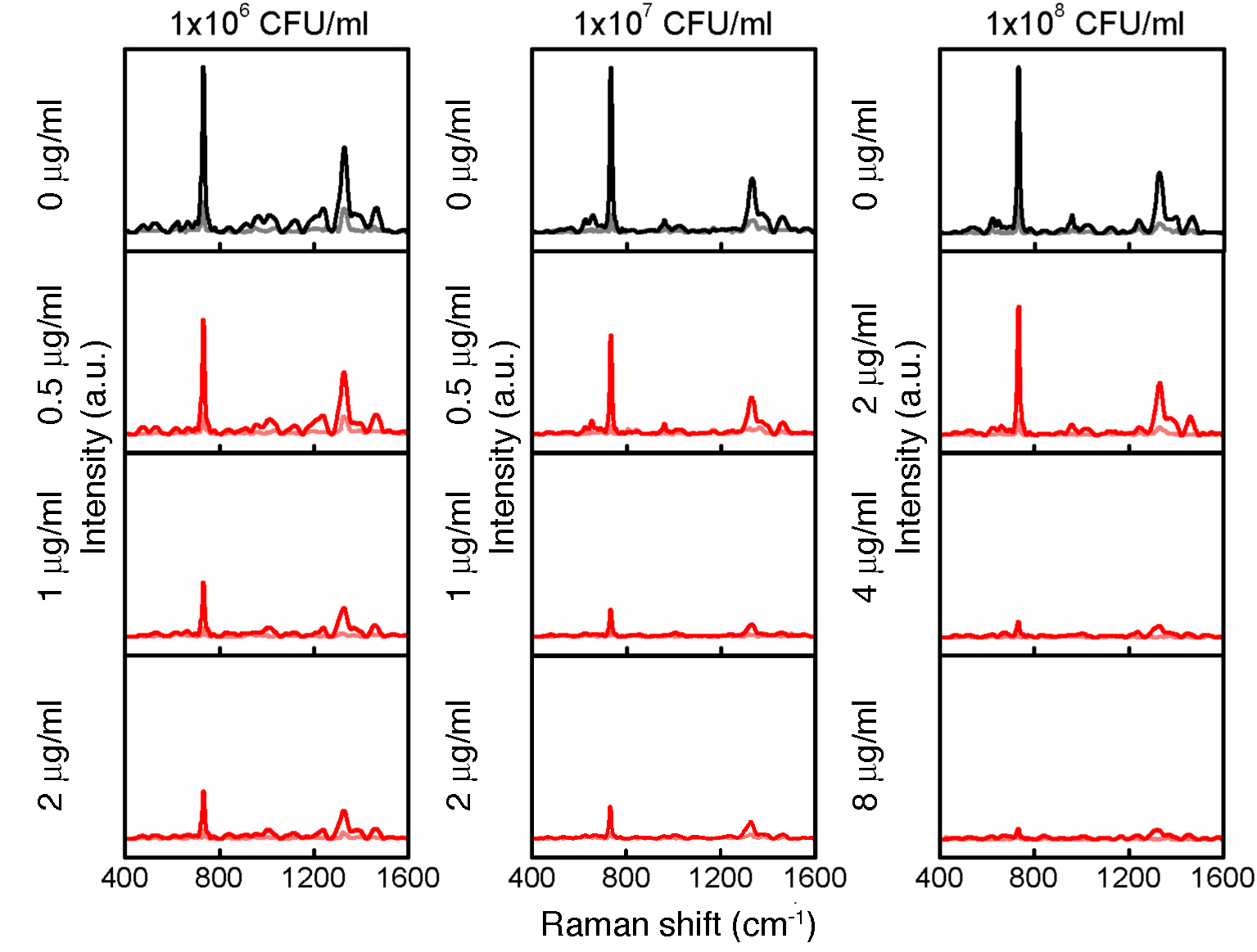


**Supplementary Figure S3 | Evolution of SERS spectra of *E. coli* versus imipenem concentration under different inoculum densities.** SERS spectra of *E. coli* (ATCC 35218) of inoculum densities of 10^6^, 10^7^ and 10^8^ CFU/ml treated with different imipenem concentrations for 2 hr. Black and red curves represent the mean SERS spectra, while gray and light red curves represent their corresponding standard deviation.


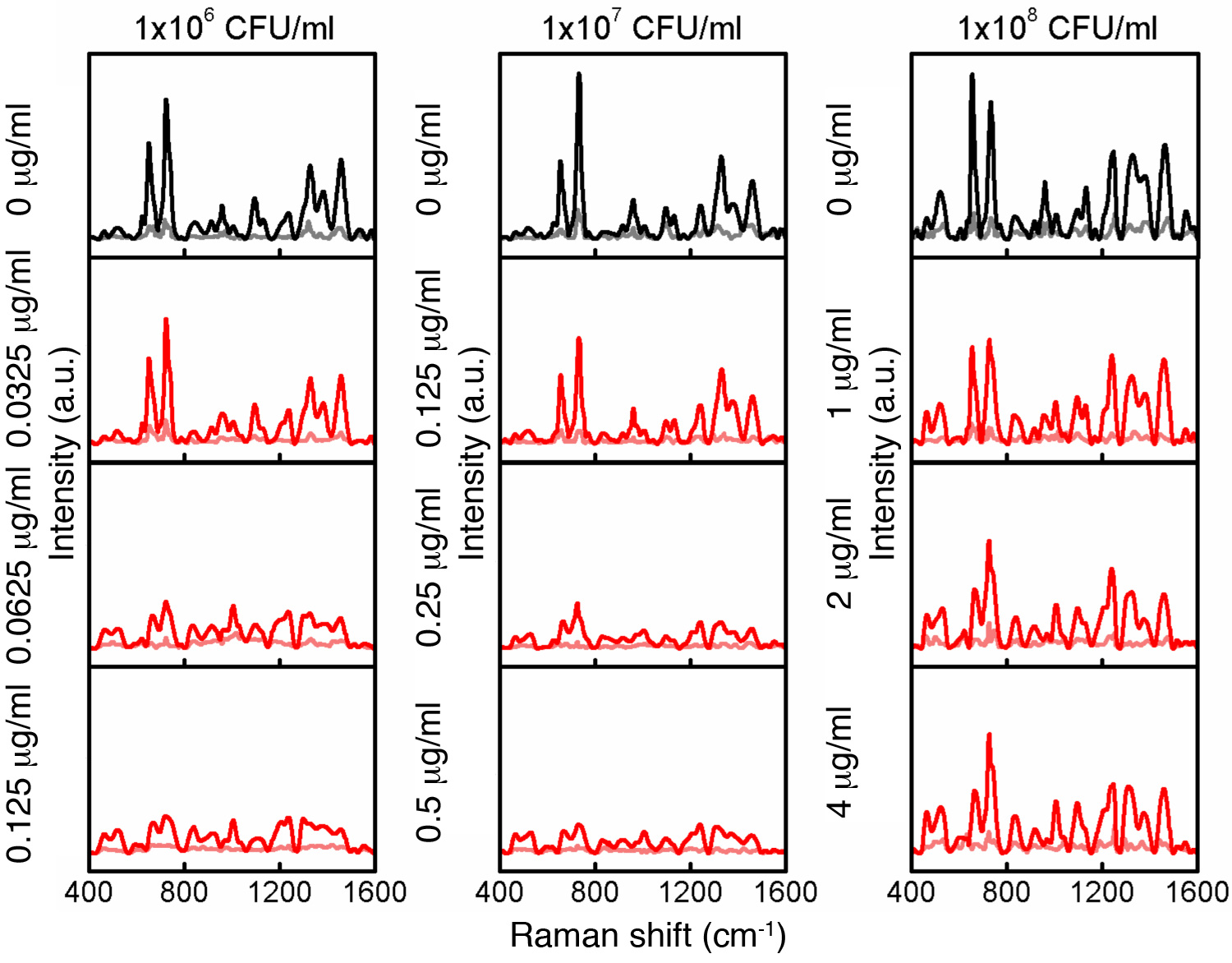


**Supplementary Figure S4 | Bright-field images of *S. aureus* of (a) 10^9^ CFU/ml (b) 10^8^ CFU/ml on a SERS substrate. Red lines in (c) and (d) are averaged SERS spectra taken from 25 different areas covered by bacteria in (a) and empty areas in (b) respectively. The beam spot size of the laser was kept below 4 μm to ensure all the 25 spectra used for (d) were taken from areas without bacteria. Black lines are typical SERS spectra of blank substrate.**


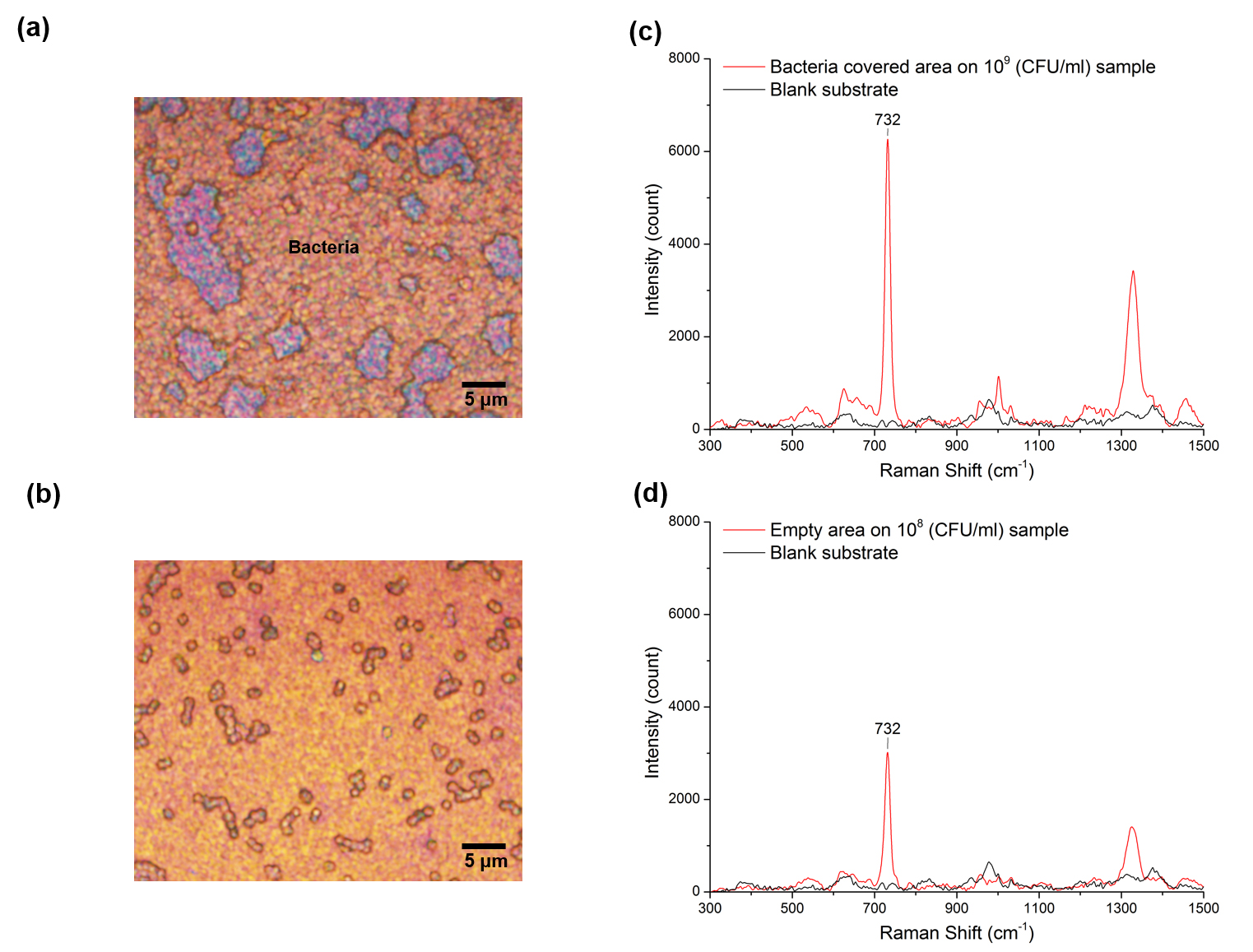


**Supplementary Figure S5 | SERS spectra of different species of (a) Gram-positive and (b) Gram-negative bacteria exhibiting the prominent 730 and 654/724 cm^-1^ peaks, respectively.**


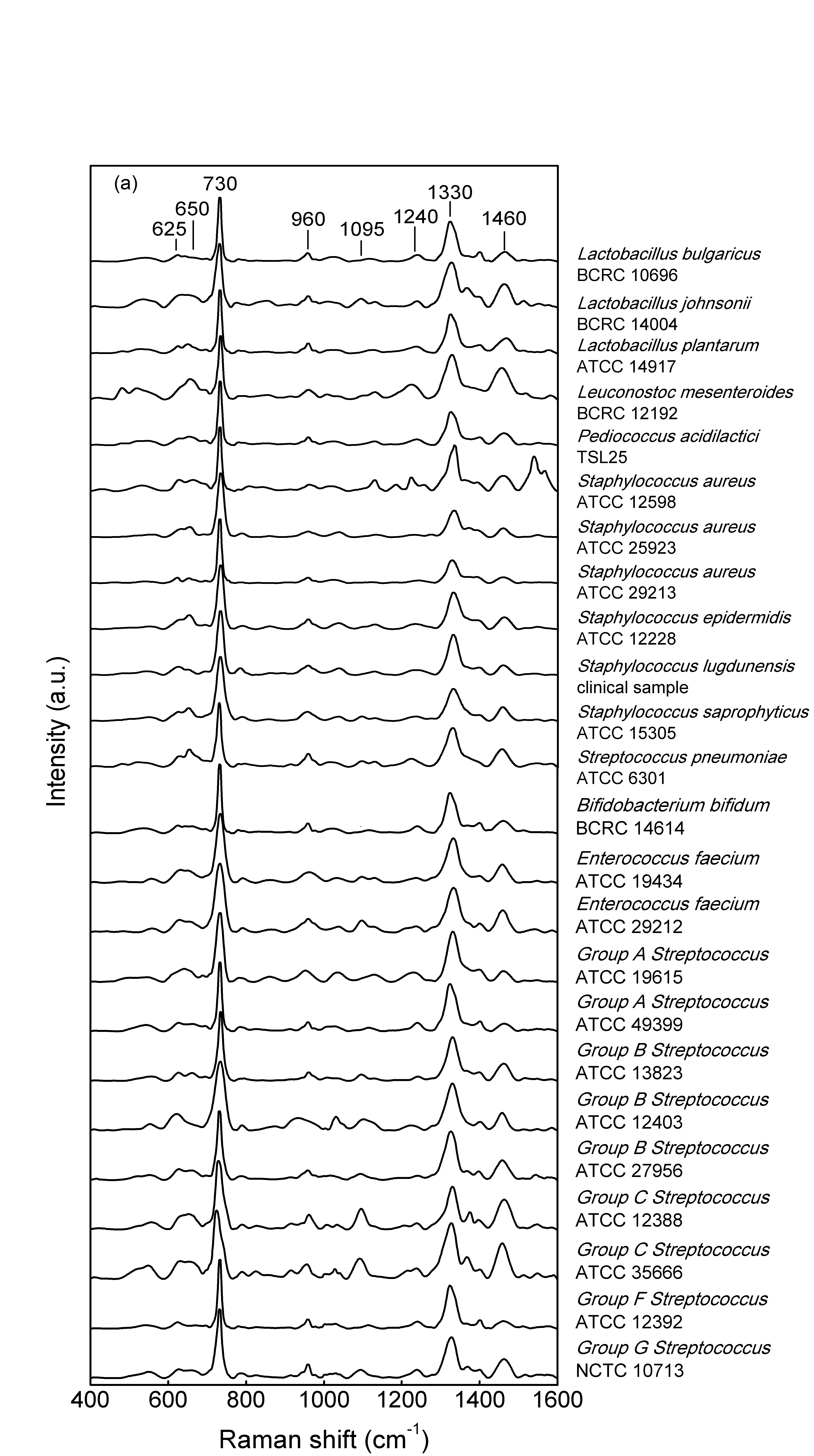

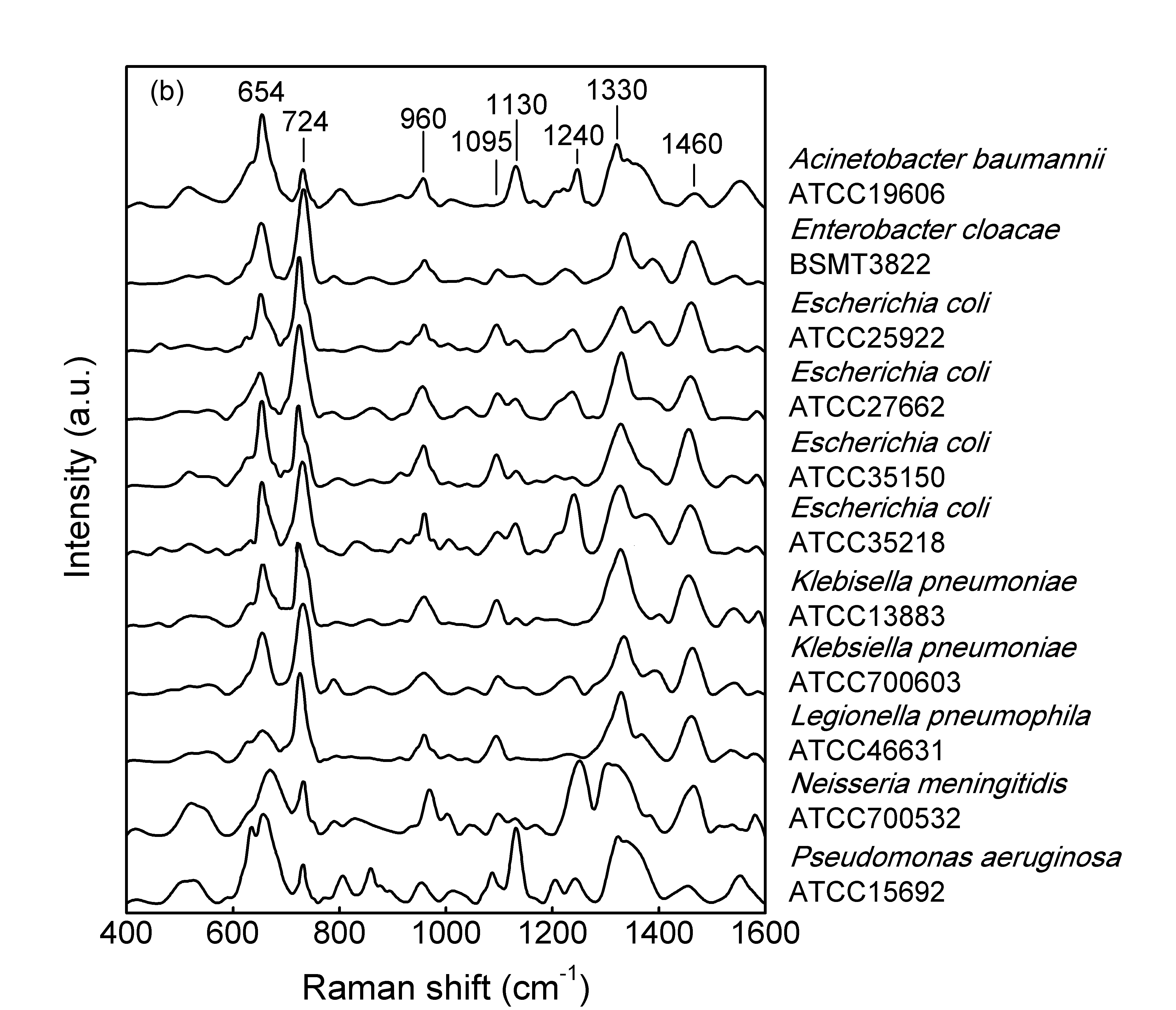

Supplement: Supplementary Information [file srep23375-s1.doc]
